# Mutations in nuclear pore complex promote osmotolerance in Arabidopsis by suppressing the nuclear translocation of ACQOS and its osmotically induced immunity

**DOI:** 10.3389/fpls.2024.1304366

**Published:** 2024-01-22

**Authors:** Kento Mori, Yusuke Murakoshi, Masashi Tamura, Satoru Kunitake, Kohji Nishimura, Hirotaka Ariga, Keisuke Tanaka, Satoshi Iuchi, Izumi Yotsui, Yoichi Sakata, Teruaki Taji

**Affiliations:** ^1^Department of Bioscience, Tokyo University of Agriculture, Tokyo, Japan; ^2^Department of Life Sciences, Faculty of Life and Environmental Sciences, Shimane University, Matsue, Japan; ^3^Department of Plant Sciences, Institute of Agrobiological Science, NARO, Tsukuba, Ibaraki, Japan; ^4^Nodai Genome Center, Tokyo University of Agriculture, Tokyo, Japan; ^5^RIKEN BioResource Research Center, Tsukuba, Ibaraki, Japan

**Keywords:** osmotolerance, nuclear pore complex, NB-LRR transport, immune response, Arabidopsis thaliana accession

## Abstract

We have previously reported a wide variation in salt tolerance among *Arabidopsis thaliana* accessions and identified *ACQOS*, encoding a nucleotide-binding leucine-rich repeat (NLR) protein, as the causal gene responsible for the disturbance of acquired osmotolerance induced after mild salt stress. *ACQOS* is conserved among Arabidopsis osmosensitive accessions, including Col-0. In response to osmotic stress, it induces detrimental autoimmunity, resulting in suppression of osmotolerance, but how *ACQOS* triggers autoimmunity remains unclear. Here, we screened *acquired osmotolerance* (*aot*) mutants from EMS-mutagenized Col-0 seeds and isolated the *aot19* mutant. In comparison with the wild type (WT), this mutant had acquired osmotolerance and decreased expression levels of pathogenesis-related genes. It had a mutation in a splicing acceptor site in *NUCLEOPORIN 85* (*NUP85*), which encodes a component of the nuclear pore complex. A mutant with a T-DNA insertion in *NUP85* acquired osmotolerance similar to *aot19.* The WT gene complemented the osmotolerant phenotype of *aot19*. We evaluated the acquired osmotolerance of five *nup* mutants of outer-ring *NUP*s and found that *nup96*, *nup107*, and *aot19/nup85*, but not *nup43* or *nup133*, showed acquired osmotolerance. We examined the subcellular localization of the GFP–ACQOS protein and found that its nuclear translocation in response to osmotic stress was suppressed in *aot19*. We suggest that NUP85 is essential for the nuclear translocation of ACQOS, and the loss-of-function mutation of NUP85 results in acquired osmotolerance by suppressing ACQOS-induced autoimmunity in response to osmotic stress.

## Introduction

Osmotic stress caused by drought, salt or cold reduces plant growth. Acquired stress tolerance is defined as the ability of plants to withstand stress following an initial stress exposure (Sung et al., 2003). Acquired osmotolerance after salt stress is widespread among *Arabidopsis thaliana* accessions (Katori et al., 2010). Pre-exposure of 7-d-old seedlings to 100 mM NaCl for 7 d (acclimation period) leads to acquired osmotolerance to 750 mM sorbitol in some *A. thaliana* accessions (Ariga et al., 2017). We have identified ACQOS as the gene responsible for acquired osmotolerance. ACQOS is identical to VICTR, which encodes a nucleotide-binding leucine-rich repeat (NLR) protein (Ariga et al., 2017). This protein interacts with PHYTOALEXIN DEFICIENT4 (PAD4) and ENHANCED DISEASE SUSCEPTIBILITY1 (EDS1) in the nucleus to activate the immune response (Kim et al., 2012). ACQOS contributes to bacterial resistance in the absence of osmotic stress, but causes detrimental autoimmunity via EDS1 and PAD4, thereby reducing osmotolerance under osmotic stress (Ariga et al., 2017). The enhanced immune response mediated by EDS1/PAD4 induces programmed cell death (PCD) (Bartsch et al., 2006). Accessions with functional *ACQOS* alleles (e.g., Col-0) are impaired in acquired osmotolerance, whereas accessions with non-functional alleles (e.g., Bu-5) show acquired osmotolerance (Ariga et al., 2017).

ACQOS induces the immune response via EDS1 and PAD4 (Kim et al., 2012; Ariga et al., 2017). EDS1 and PAD4 localize in the nucleus, while NLRs localize in the cytoplasm and nucleus (Zhu et al., 2010; Alcázar and Parker, 2011; Roth et al., 2017; Zhang et al., 2020). The mechanism of ACQOS translocation to the nucleus is unknown. The nuclear pore complex (NPC) is responsible for the nuclear–cytoplasmic transport of mRNA and proteins (Wiermer et al., 2012). It allows cytoplasmic-to-nuclear transport of proteins larger than 40–60 kDa through importins and exportins (Wang and Brattain, 2007; Saier et al., 2014). The NPC consists of about 30 different nucleoporins (Nups) (Wälde and Kehlenbach, 2010), which form an outer cytoplasmic region and an inner nucleoplasmic region. The symmetrical core region consists of outer ring-, linker-, inner ring-, transmembrane ring-, and central phenylalanine-glycine NUPs (Yang et al., 2017). Abscisic acid (ABA) is important in osmotic stress responses, and its enhanced signaling improves osmotic tolerance (Nakashima et al., 2009). A mutation in an NPC component, *HIGH EXPRESSION OF OSMOTICALLY RESPONSIVE GENES1* (*HOS1*) increases transcript levels of cold-responsive genes such as *RESPONSIVE TO DESICCATION 29A* (*RD29A*) and *COLD-REGULATED 15A* (*COR15A*) under cold stress relative to WT (Ishitani et al., 1998). On the other hand, both *hos1* and *nup85* mutations suppress their expression under ABA, salt, and osmotic stress (Zhu et al., 2017). NUP85 interacts with the transcription factor MED18, which controls the expression of osmotic stress–responsive genes (Lai et al., 2014; Liao et al., 2016) In both mutants, sensitivity to ABA and mild salt stress is higher than in WT (Zhu et al., 2017). The double mutant *bak1 bkk1*, a co-receptor of the brassinosteroid (BR) receptor BRI1, BRI1-ASSOCIATED KINASE 1 (BAK1), and its homolog BAK1-LIKE 1 (BKK1), exhibits a salicylic acid-dependent cell death phenotype and constitutive expression of *PR* genes even without pathogen invasion (He et al., 2007). *NUP85* was identified as the causal gene for a *suppressor of bak1 bkk1* (*sbb1-1*) mutant, which suppresses the cell death phenotype of *bak1 bkk1* (Du et al., 2016). It suggests that NUP is also involved in plant defense and the regulation of cell death.

*Suppressor of npr1-1, constitutive 1* (*snc1*) is a gain-of-function Arabidopsis mutant carrying a mutation in *NLR* that constitutively activates resistance responses to pathogens (Li et al., 2001; Zhang et al., 2003). *Modifiers of snc* (*mos*) *1*, *3*, and *6* suppress the autoimmune responses mediated by *snc1* (Palma et al., 2005; Zhang and Li, 2005). *MOS3* encodes NUP96 and *MOS6* encodes importin α3. Nuclear accumulation of SNC1 is inhibited in those mutants, which explains the suppression of the autoimmune responses (Zhu et al., 2010; Roth et al., 2017; Zhang et al., 2020). In addition to *ACQOS*, mutations in *EDS1* and *PAD4* (Alcázar and Parker, 2011; Cui et al., 2017), as well as *RAR1* and *SGT1*, whose products facilitate stable NLR protein accumulation and function (Takahashi et al., 2003) result in acquired osmotolerance in the Col-0 background (Ariga et al., 2017). However, the molecules involved in the localization of ACQOS are unknown.

Here, we EMS-mutagenized Col-0 seeds, isolated *acquired osmotolerance* (*aot*) mutants. We sequenced 5 genes including *ACQOS*, *EDS1*, *PAD4*, *RAR1*, and *SGT1* known to be involved in acquired osmotolerance in the Col-0 background (Ariga et al., 2017) and characterized one of the candidate mutants that none of these genes was mutated.

## Materials and methods

### Plant materials and growth conditions

*Arabidopsis thaliana* seeds were sown on agar (1.0% w/v) plates containing full-strength Murashige and Skoog (MS) salts with a vitamin mixture (10 mg L^−1^ myoinositol, 200 µg L^−1^ glycine, 50 µg L^−1^ nicotinic acid, 50 µg L^−1^ pyridoxine hydrochloride, 10 µg L^−1^ thiamine hydrochloride, pH 5.7) and 1% w/w sucrose. Plates were sealed with surgical tape, the seeds were stratified at 4°C for 4 to 7 d, and transferred to a growth chamber (80 µmol photons m^2^ s^−1^; 16/8-h light/dark cycle; 22°C) for germination and growth (Isono et al., 2020).

Seeds of the *A. thaliana* Be-1 and Col-0 background mutants *nup85-2* (SALK_113274), *nup43* (SALK_095344C), *mos3-2/nup96* (CS69987), *nup107* (SALK_057072C), *nup133* (SALK_092608C), and *mos6* (SALK_119474C) were obtained from the *Arabidopsis* Biological Resource Center (ABRC, Ohio State University).

### Mutagenesis and *aot* mutant screening

EMS-mutagenesis was performed as described in Isono et al. (2020). Acquired osmotolerance assay: Seedlings (7-d old) were grown on nylon mesh (990 μm) on an MS agar plate supplemented with 100 mM NaCl for 7 d and were then mesh-transferred to a plate supplemented with 750 mM sorbitol for 15 d or 38 d. Though Col-0 WT plants cannot survive in this assay, plants that survive under these conditions were isolated as aot candidates.

### Abiotic stress assays

Seedlings (10-d old) were grown on nylon mesh (990 μm) on an MS agar plate supplemented with 600 mM sorbitol for 16 d (osmotic-shock stress) or 225 mM NaCl for 8 d (salt-shock stress). Their chlorophyll content was determined according to Porra et al. (1989).

### RNA extraction, RT-PCR and qRT-PCR

Total RNA extraction and RT-PCR or qRT-PCR analysis were performed as described in Isono et al. (2020) (Isono et al., 2020). *ACTIN2* was used as the internal standard for qRT-PCR. The primers are listed in **Supplementary Table S1**.

### Genetic mapping of the causative gene of the *aot19* phenotype

We crossed the *aot19* mutant with Be-1, an accession that does not acquire osmotolerance like Col-0, and selfed the resulting F_1_ progeny to generate an F_2_ population. Genomic DNA was prepared from individual F_2_ plants with the osmotolerant phenotype and used as a PCR template. The simple-sequence-length polymorphism markers for mapping are listed in **Supplementary Table S1**. PCR conditions were as follows: initial denaturation at 94°C for 2 min; 34 cycles at 94°C for 20 s, 56 to 59°C for 20 s, and 72°C for 20 s; and final extension at 72°C for 2 min. Microsatellites were fractionated in 5% to 7% agarose gel, and recombination frequencies (%) were calculated from the band pattern.

### DNA library construction, whole-genome sequencing of the *aod19* mutant, and detection of mutations

All procedures were performed as in Tsukimoto et al. (2021). The read data were submitted to the DNA Data Bank of Japan Sequence Read Archive (accession number DRA016084).

### Plasmid construction and transformation

For complementation test, the genomic region of *At4g32910* (793 bp upstream of the ATG initiation codon to 690 bp downstream of the terminator) derived from WT Col-0 was amplified by PCR with *AOT19/NUP85* primers and cloned into the binary vector pGreen0029. The construct was introduced into *Agrobacterium tumefaciens* GV3101, which was used for plant transformation to *aot19* by the floral dip method. Transgenic plants were selected on MS agar plates containing 200 µg ml^−1^ claforan and 25 µg ml^−1^ kanamycin. Ten-day-old seedlings (T_1_ plants) were transferred to soil in pots.

For construction of *p35S:mGFP-ACQOS*, to avoid GFP dimerization, a A207K substitution (Zacharias et al., 2002), which eliminates the dimerization, was introduced into the *sGFP* gene of pGH35S-sGFP (Fujita et al., 2009) and the construct obtained was named pGH35S-mGFP. To obtain the coding sequence (CDS) of *ACQOS*, six exons of *At5g46520* were independently amplified and then joined by overlapping-PCR. Using ACQOS_CDS_F and ACQOS_CDS_R primers, the final CDS was amplified without a stop codon and ligated into the *EcoR*V site of the pGH35S-mGFP vector. All primers are listed in **Supplementary Table S1**.

### Cell death detection by trypan blue staining

Trypan blue was dissolved in a mixture of equal volumes of lactic acid (85% w/w), phenol (TE buffered, pH 7.5–8.0), glycerol, and distilled water in a plastic test tube. Seedlings were immersed in fresh trypan blue solution (0.2% w/v), boiled in a water bath for 1 min, incubated for 3 h at room temperature, and destained in chloral hydrate solution (500 g of chloral hydrate dissolved in 195 ml of distilled water) for 2 d. Destained seedlings were mounted in glycerol solution (70% v/v) for microscopy. Photographs were taken under a microscope (SZ61 or DP22, Olympus, Tokyo) under white light.

### Microscopic analysis

Transgenic seedlings (4-day old) grown on an MS agar plate were transferred to a plate supplemented with 600 mM sorbitol for 2 d (osmotic-shock stress). Nuclei in the seedlings were stained in PBS containing 0.5 μg/ml Hoechst 33342 for 20–30 min before fluorescence microscopy. Images were obtained with an all-in-one fluorescence microscope (BZ-X800, Keyence, Osaka, Japan) equipped with an optical sectioning module and a Plan Apochromat 40× objective (NA0.95 and BZ-PA40, respectively, both from Keyence, Tokyo). Green fluorescence was detected with a GFP filter (ex 470/40 nm, em 525/50 nm, dichroic 495 nm; OP-87763, Keyence). Blue fluorescence (Hoechst 33342) was detected with a DAPI filter (ex 360/40 nm, em 460/50 nm, dichroic 400 nm; OP-87762, Keyence).

## Results

### Isolation of the acquired osmotolerance 19 mutant

**Figure 1:**
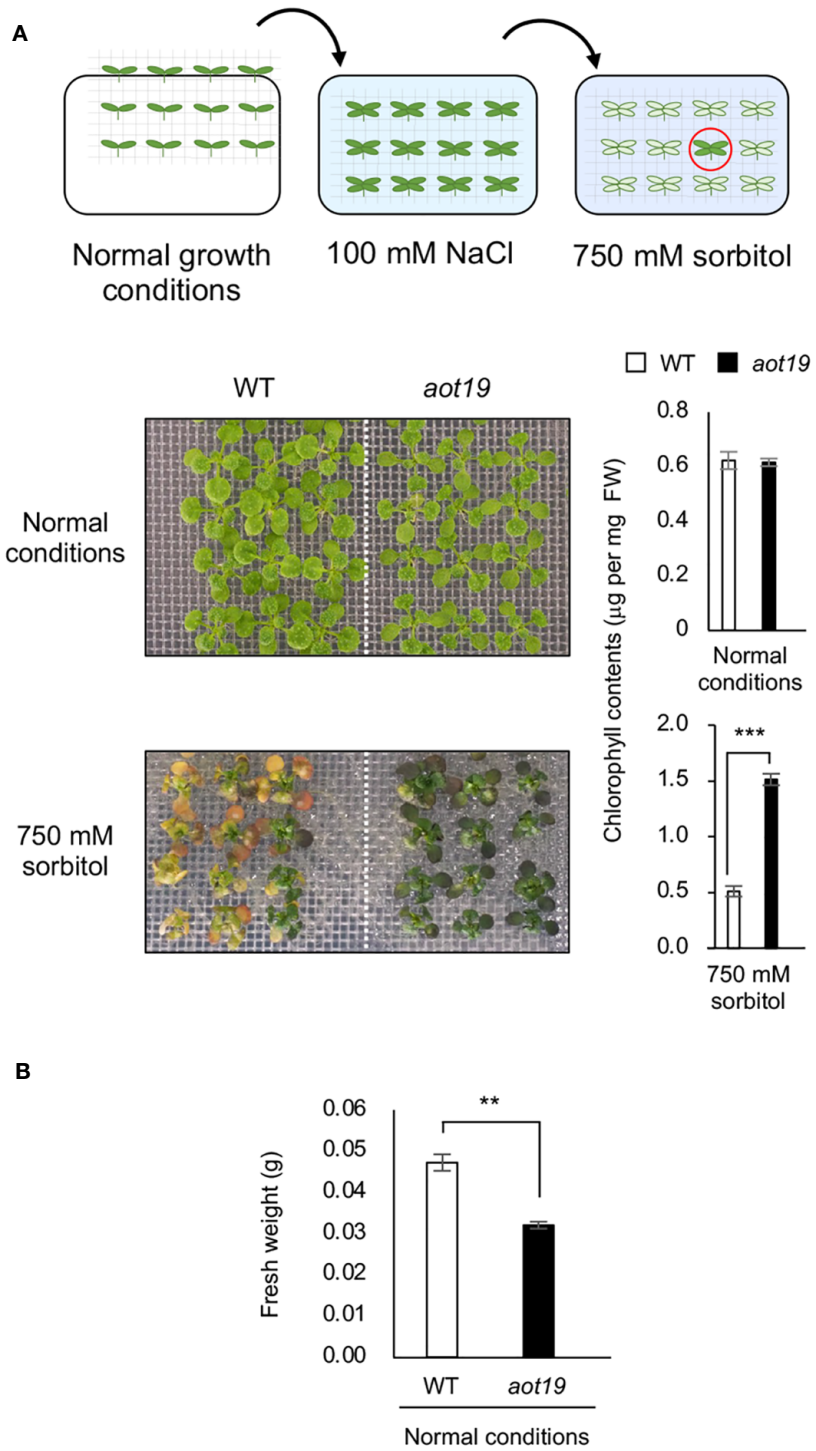
Identification of the *acquired osmotolerance* (*aot*) *19* mutant. **(A)** Flow chart of the acquired osmotolerance assay. A total of 19 500 EMS-mutagenized, salt-acclimatized, 14-d-old seedlings of accession Col-0 were transferred to Murashige and Skoog (MS) agar plates containing 750 mM sorbitol for 15 d, and osmotolerant seedlings (red circle) were selected. Upper photos: 14-day-old wild-type (WT) and *aot19* seedlings grown under normal conditions. Lower photos: acquired osmotolerance of the WT and *aot19* plants. Right panels: chlorophyll content as an index of acquired osmotolerance. FW, fresh weight. **(B)** Fresh weight of plants grown as described in *A* under normal growth conditions. Differences between WT and *aot19* were analyzed by Student’s *t*-test (mean ± SE, *n* = 3, **P < 0.01, ***P < 0.001).

We screened 19 500 M_2_ seedlings from an EMS-mutagenized Col-0 population for acquired osmotolerance to severe osmotic stress (750 mM sorbitol) after mild-salt acclimatization (100 mM NaCl for 7 d) and isolated 13 *aot* mutant candidates. We sequenced 5 genes known to be involved in acquired osmotolerance in the Col-0 background (Ariga et al., 2017) and detected mutations in *ACQOS* (7 lines), *EDS1* (3 lines), *PAD4* (1 line), and *RAR1* (1 line), but no mutations in *SGT1* (data not shown), but none of these genes was mutated in *aot19*. WT Col-0 did not acquire osmotolerance, but *aot19* did (**Figure 1A**). Chlorophyll content did not differ significantly between WT and *aot19* under normal conditions, but was significantly higher in *aot19* after exposure to 750 mM sorbitol (**Figure 1A**). The fresh weight of *aot19* plants tended to be smaller than that of WT plants under normal growth conditions (**Figure 1B**).

### Characterization of the *aot19* mutant

**Figure 2:**
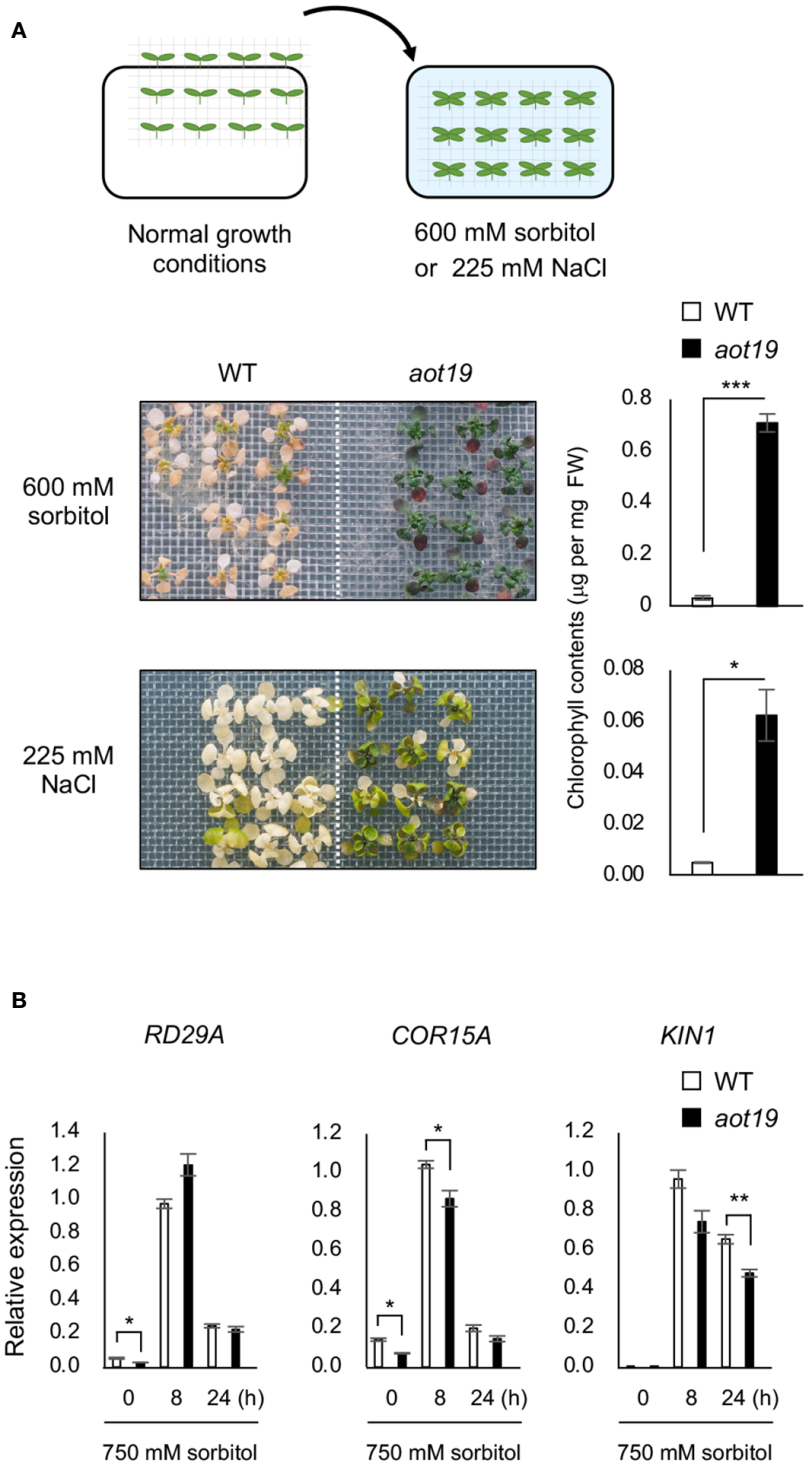
Characterization of the *aot19* mutant. **(A)** Top: Flow chart of the salt- and osmo-shock tolerance assay. Seedlings (14-d old) were transferred to MS agar plates containing 225 mM NaCl for 8 d or 600 mM sorbitol for 16 d. Upper photos: osmo-shock tolerance of the WT and *aot19* plants. Lower photos: salt-shock tolerance of the WT and *aot19* plants. Right panels: Chlorophyll content of the salt- and osmo-shock tolerances. **(B)** Expression of osmostress-responsive marker genes in WT and *aot19* under normal conditions and acquired osmotic stress (100 mM NaCl for 7 d and subsequent 750 mM sorbitol for 8 or 24 h). Expression levels were determined by quantitative real-time polymerase chain reaction (qRT-PCR) and were normalized to those of *Actin2* (mean ± SE, *n* = 3). Differences between WT and *aot19* were analyzed by Student’s *t*-test (mean ± SE, *n* = 3, *P < 0.05, **P < 0.01, ***P < 0.001).

The *aot19* plants were directly exposed to osmotic or salt stress without salt acclimatization (**Figure 2A**). Tolerance to osmo-shock (600 mM sorbitol for 16 d) and salt-shock (225 mM NaCl for 8 d) stresses was higher in *aot19* than in WT plants (**Figure 2A**), suggesting that *AOT19* negatively regulates acquired osmotolerance and osmo-shock tolerance. The mRNA levels of the ABA/osmostress marker genes *RD29A*, *COR15A*, and *KIN1* were comparable between WT and *aot19*, although they were rather lower in *aot19* than in WT at some time point under osmotic stress (**Figure 2B**), indicating that the osmotolerance of *aot19* was not due to an increase in osmotic- and ABA-responsive gene expressions.

### Identification of the causal gene in *aot19*

**Figure 3:**
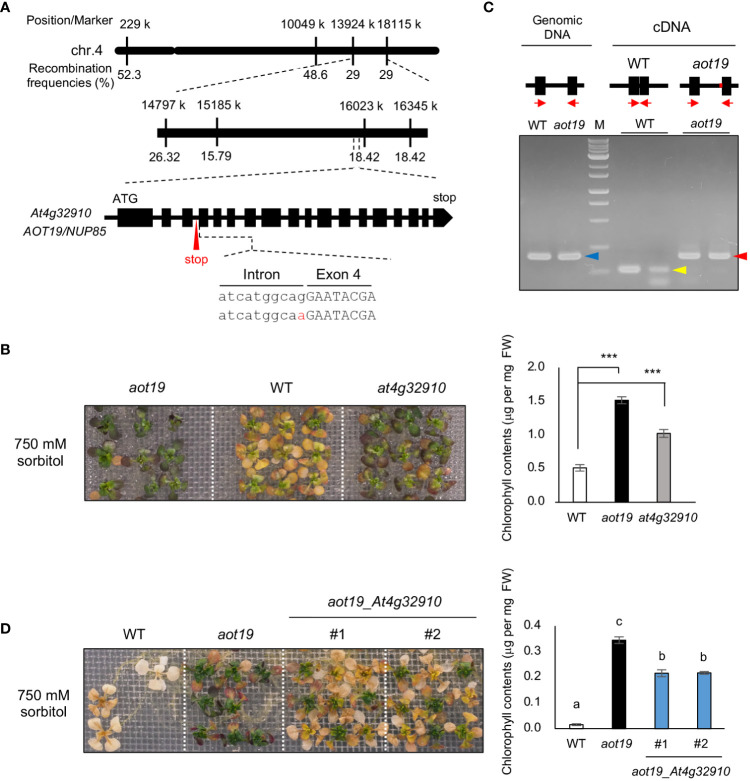
Identification of the causal gene in *aot19*. **(A)** High-resolution mapping of the causal locus in *aot19* using F_2_ progeny between *aot19* and Be-1. A mutation in *aot19* (red) results in intron retention and a stop codon (red arrow). **(B)** Acquired osmotolerance assay of *aot19*, WT, and a T-DNA insertion mutant of *At4g32910* in WT (*at4g32910*). The assay was performed as in **Figure 1A**. Photo: acquired osmotolerance of *aot19*, WT, and *at4g32910* plants. Right panel: chlorophyll contents of the seedlings. Differences between the WT and the mutants were analyzed by Student’s *t*-test (mean ± SE, *n* = 3, ***P < 0.001). **(C)** Detection of intron retention by PCR. Upper panel: The block and line mean exon and intron, respectively. The red on the line means the mutation occurred in *aot19*. The red arrows indicate the positions of primers. Genomic PCR (blue arrow) and RT-PCR in *aot19* (red arrow) and WT (yellow arrow) with primers designed for the exons before and after the mutation (*n* = 2). **(D)** Complementation test of *aot19* with *At4g32910*. T_2_ plants transformed with their native promoter: *At4g32910* (*aot19*_ *At4g32910*) derived from the WT Col-0 were used. The salt -acclimated plants were mesh-transferred to a plate supplemented with 750 mM sorbitol for 38 d. Right panel: chlorophyll contents of the seedlings. Bars labeled with different letters differed significantly (*P* < 0.05, one-way ANOVA with *post hoc* Tukey HSD test, mean ± SE, *n* = 3).

High-resolution mapping using the F_2_ progeny from a cross between Be-1 and *aot19* plants revealed the locus responsible for the osmotolerance of *aot19* on chromosome 4 near position 16 000 k within a 900-kbp region (**Figure 3A**). Whole-genome sequencing revealed non-synonymous mutations in three genes within this region. We examined the acquired osmotolerance of the T-DNA insertion mutants of these genes that obtained from ABRC and found that only the *at4g32910* mutant exhibited the osmotolerance compared with WT plants (**Figure 3B**; **Supplementary Figure S1**). In *aot19*, there is a mutation at the acceptor site of the third intron of the *At4g32910* gene, resulting in intron retention (**Figure 3A**). The intron retention was confirmed by RT-PCR with primers designed for the exons adjacent to or surrounding the mutation (**Figure 3C**). It resulted in a stop codon within the intron, presumably resulting in a truncated At4g32910 protein (**Figure 3C**). In a complementation test, the *aot19* plants transformed with *At4g32910* (with its native promoter) had impaired acquired osmotolerance; chlorophyll content was significantly lower in these lines than in *aot19* (**Figure 3D**). These results suggest that *At4g32910* is the causal gene for *aot19*. *AOT19* is identical to *NUCLEOPORIN 85* (*NUP85*) (**Figure 3A**) and encodes a nuclear pore complex (NPC) component.

### Analysis of AOT19/NUP85 function for osmotolerance

**Figure 4:**
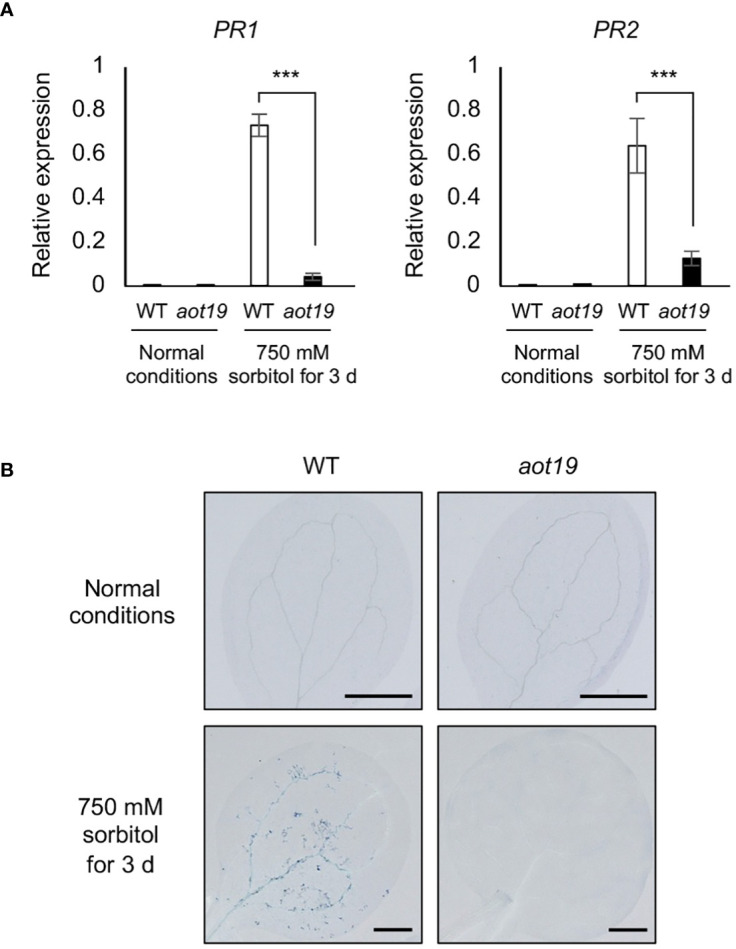
Immune response and programmed cell death in *aot19*. **(A)** Expression of pathogenesis-related (*PR*) *1* and *PR2* genes in WT and *aot19* plants under normal and acquired osmotolerance conditions (100 mM NaCl for 7 d followed by 750 mM sorbitol for 3 d). Expression levels were determined by qRT-PCR and were normalized to those of *Actin2*. Differences between WT and *aot19* plants were analyzed by Student’s *t*-test (mean ± SE, *n* = 3, ***P < 0.001). **(B)** Trypan blue staining of seedling leaves.

To investigate whether *aot19* suppresses the osmostress-induced immune responses, we analyzed the expression levels of *PATHOGENESIS-RELATED* (*PR*) genes. Under osmotic stress (750 mM sorbitol for 3 d), expression of the *PR1* and *PR2* genes increased in WT, whereas this increase was significantly suppressed in *aot19* (**Figure 4A**). We examined the expression levels of osmotic- and ABA-responsive genes *RD29A*, *COR47*, *COR15A* and *ABI5*, in addition to immune responsive genes *PR1*, *PR2* and *PR5* under mild and severe osmotic stress (**Supplementary Figure S2**). There were no substantial differences in osmotic- and ABA-responsive gene expressions between WT and *aot19* mutants under either osmotic stress, although they were lower in *aot19* than in WT at some point. As for the expression levels of *PR1* and *PR2*, little expression was observed in both WT and *aot19* mutant under mild osmotic stress. However, under severe osmotic stress, the expression levels of *PR1*, *PR2* and *PR5* increased significantly in WT compared with *aot19*. These results suggest that the mechanism by which *aot19* exhibits osmotolerance is not due to an increase in osmotic- and ABA-responsive gene expressions, but rather to suppression of the enhanced immune response under severe osmotic stress.

We investigated whether PCD may be suppressed in rosette leaves of *aot19* under osmotic stress. Osmotic stress increased trypan blue staining in WT leaves, but *aot19* leaves were scarcely stained under either normal or osmotic-stress conditions (**Figure 4B**), suggesting that AOT19/NUP85 regulates osmostress-induced PCD.

**Figure 5:**
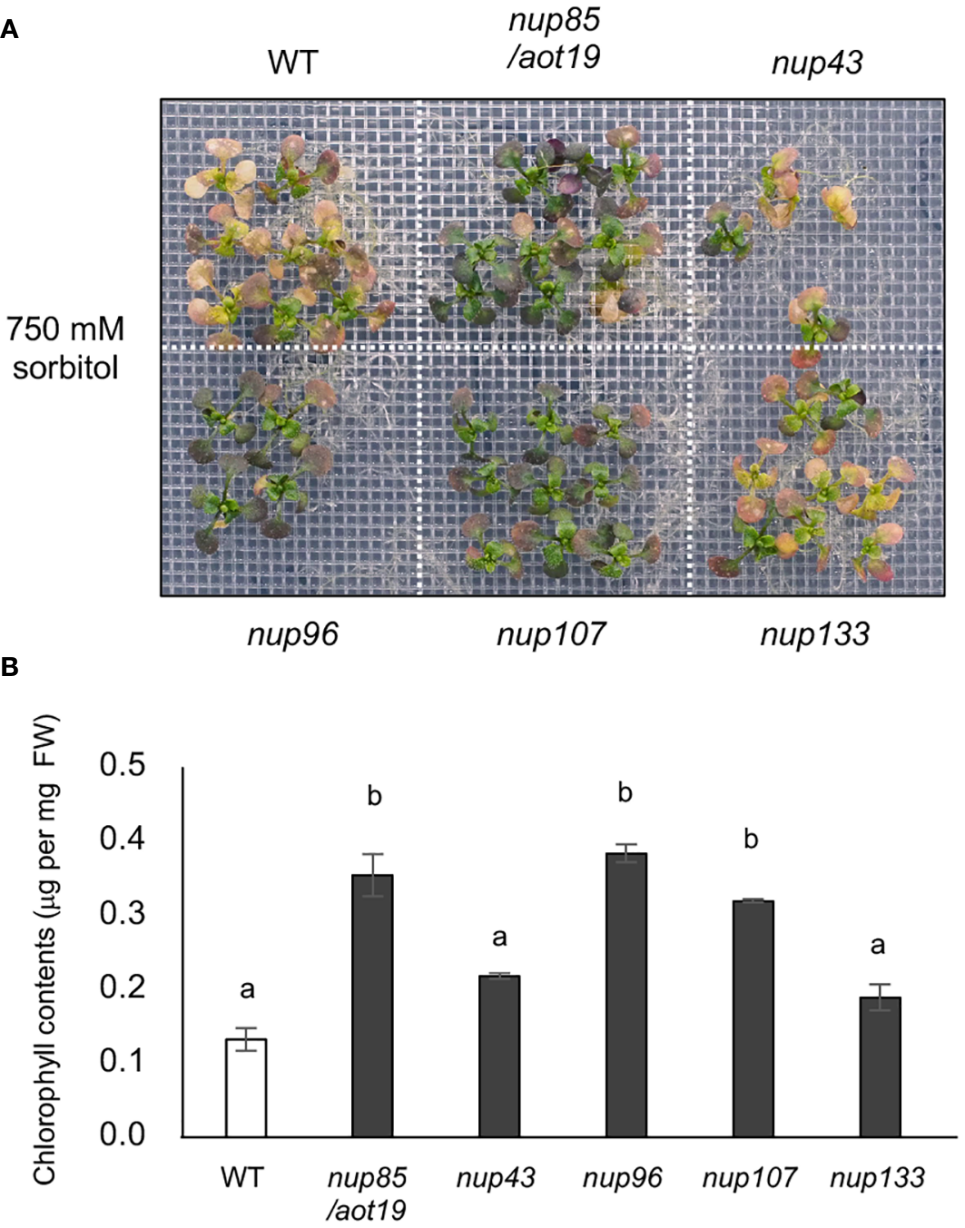
Acquired osmotolerance of *nup* mutants. **(A)** Acquired osmotolerance of Col-0 WT and Col-0-background *aot19/nup85*, *nup43*, *nup96*, *nup107*, and *nup133* mutants. **(B)** Chlorophyll contents of the seedlings shown in **(A)** Bars labeled with different letters differed significantly (*P* < 0.05, one-way ANOVA with *post hoc* Tukey HSD test, mean ± SE, *n* = 3).

We evaluated the acquired osmotolerance of five *nup* mutants of outer-ring NUPs and found that *nup96*, *nup107*, and *aot19/*nup85, but not *nup43* or *nup133*, showed acquired osmotolerance (**Figure 5**; **Supplementary Figure S1**). These findings suggest that mutations in some NPC components suppress acquired osmotolerance in Col-0.

### Subcellular localization of ACQOS in *aot19*

**Figure 6:**
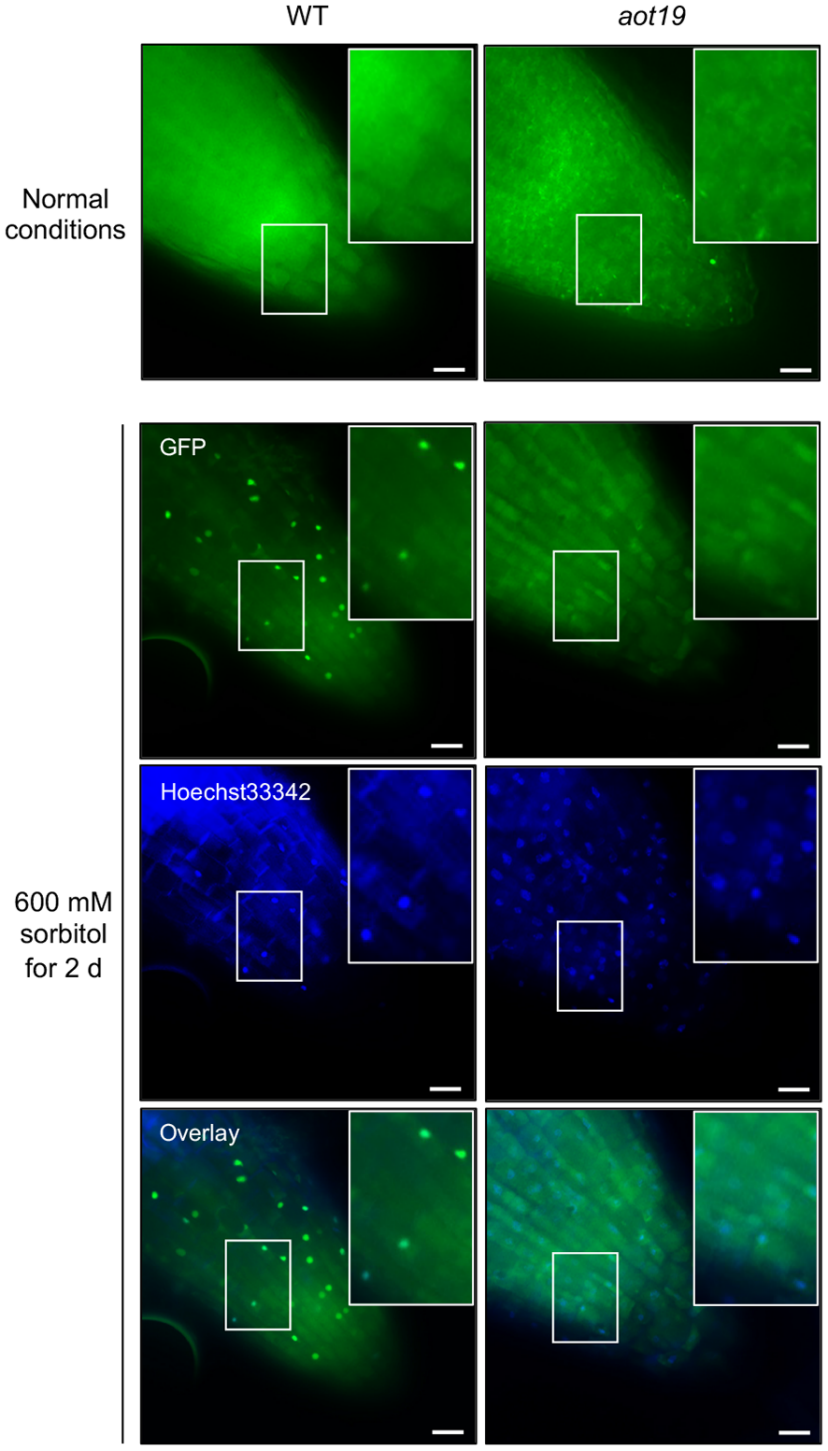
Subcellular localization of mGFP-ACQOS in roots. Seedlings of transgenic Arabidopsis Col-0 plants stably expressing ACQOS fused to the C-terminus of monomeric green fluorescent protein (mGFP) under the control of the promoter of the cauliflower mosaic virus (CaMV) 35S gene (*p35S:mGFP-ACQOS*). Seedlings were grown under normal conditions or osmotic stress (600 mM sorbitol for 2 d). Single-section images of the GFP channel (top), nucleus channel showing staining with Hoechst 33342 (middle), and their overlay (bottom) are shown in each panel. In each image, a large rectangle shows an enlargement of the area enclosed by the small rectangle. Scale bars, 20 μm.

We hypothesized that ACQOS is unable to translocate from the cytoplasm into the nucleus in *aot19*. To test this hypothesis, we examined the subcellular localization of a GFP-ACQOS chimera under osmotic stress. In WT, ACQOS translocates from cytoplasm into nucleus in response to osmotic stress, whereas less osmostress-triggered nuclear translocation was not observed in *aot19* than in WT (**Figure 6**). These results suggest that the *AOT19/NUP85* mutation in *aot19* prevents the nuclear translocation from the cytoplasm of ACQOS under osmotic stress; therefore, the immune response mediated by the nucleus-localized immune-regulators EDS1 and PAD4 is interrupted, resulting in enhanced osmotolerance.

## Discussion

Here we report the *A. thaliana aot19* mutant that acquired osmotolerance despite the Col-0 background. This mutant carries the functional *ACQOS* allele, which encodes an NLR protein and suppresses osmotolerance owing to detrimental autoimmunity via EDS1/PAD4. An *AOT19/NUP85* mutation in *aot19* inhibited nuclear translocation of ACQOS in response to osmotic stress. We suggest that the *aot19* mutant acquires osmotolerance by suppressing the immune response due to the defect in ACQOS nuclear translocation, which is essential for ACQOS function to trigger the immune response via interaction with nuclear-localized EDS1 and PAD4 (Zhu et al., 2010; Alcázar and Parker, 2011; Roth et al., 2017; Zhang et al., 2020).

Nuclear translocation of proteins larger than 40–60 kDa requires an importin (Stewart, 2007; Wang and Brattain, 2007; Beck and Hurt, 2017). Importin MOS6 supports the nuclear translocation of the NLR-protein SNC1 (Palma et al., 2005; Roth et al., 2017; Lüdke et al., 2021). In response to osmotic stress, ACQOS (137 kDa) translocated into the nucleus in WT plants, so a yet-unidentified importin might be involved. It may be useful to examine the osmotolerance of importin knockout mutants or to isolate proteins that interact with ACQOS through the use of co-immunoprecipitation.

In *aot19*, the *AOT19/NUP85* mutation may also affect the transport of other nuclear-localized proteins. The *nup85* mutant of *Lotus japonicus* produces fewer seeds (Saito et al., 2007), as similarly seen in the *aot19* mutant. In addition, *aot19* mutant tended to have lower fresh weight than the WT plants, suggesting that the *AOT19/NUP85* mutation impacts growth and reproduction. However, the impact on growth and reproduction was not severe, perhaps because the *NUP85* mutation does not completely inhibit the cytoplasmic-to-nuclear transport of ACQOS and other proteins in *aot19*. ACQOS was detected in the nucleus in *aot19*, although less than in WT. The osmotolerance of F_1_ seedlings from the cross between Col-0 and NIL-Bu-5, which carried non-functional *ACQOS* alleles from Bu-5 in the genetic background of Col-0, was intermediate between those of Col-0 and NIL-Bu-5 (Ariga et al., 2017). This suggests that ACQOS decreases acquired osmotolerance in a protein level–dependent manner. Therefore, osmotolerance can be improved by partial limitation of ACQOS nuclear translocation.

*NUP85* has been identified as the causal gene for a mutant with a decreased expression of an abiotic stress–responsive luciferase reporter (*RD29A-LUC*) in response to ABA and salt stress (Zhu et al., 2017). The expression of ABA- and salt stress–inducible genes is lower in the *nup85* mutant than in WT, resulting in higher sensitivity of the mutant to ABA and mild salt stress of 100 mM NaCl (Zhu et al., 2017). On the other hand, *aot19/nup85* was highly tolerant to severe osmotic (600 mM sorbitol) and salt (225 mM NaCl) stresses in this study. The improved osmotolerance of *aot19* to severe osmotic stress may depend on whether ACQOS translocates to the nucleus at a particular stress intensity. Under mild osmotic stress, little expression of *PR1*, *PR2* and *PR5* was observed in both WT and *aot19* mutants, but under severe osmotic stress, the expression levels were significantly increased in WT compared to *aot19*. Thus, under severe salt and osmotic stress, *aot19/nup85* suppresses the excessive immune response by inhibiting the nuclear translocation of ACQOS, resulting in its higher tolerance than that of WT plants.

Of the *nup* mutants tested, *nup96*, *nup107*, and *aot19/nup85*, but not *nup43* or *nup133*, acquired osmotolerance. These findings suggest that NUP85, NUP96, and NUP107 are important for ACQOS nuclear translocation. These NUPs are components of the outer NPC, and NUP96 is important in the nuclear migration of SNC1. NUP85 and NUP96 affect the expression of auxin, ABA, and osmotic stress–responsive genes (Zhu et al., 2017; Zhang et al., 2020). Despite reports that the *NUP85* mutation results in decreased expression of osmotic stress-responsive genes, no substantial differences in expression levels between WT and *aot19* mutant were observed. The T-DNA mutant of *NUP85* exhibited the osmotolerance compared with WT plants. However, the *aot19* mutant was more tolerant to osmotic stress than the T-DNA mutant. In addition, the complementary line *aot19_At4g32910* impaired acquired osmotolerance compared to *aot19*, but more tolerant than WT. This may be because the immature truncated protein from *aot19* competes with the correct protein from *At4g32910*, and as a result, the expressions of osmotic stress-responsive genes are not reduced in *aot19*. The Arabidopsis genome encodes approximately 150 NLRs, but little is known about their nuclear translocation. These NUPs may be important not only in immune responses but also in various environmental responses via nuclear translocation of NLRs.

## Conclusion

We isolated the *aot19* mutant, which shows acquired osmotolerance despite its Col-0 background and the presence of functional *ACQOS* alleles. The causal gene of *aot19* is identical to *NUP85*, which encodes an NPC component. In *aot19*, the nuclear translocation of ACQOS and detrimental autoimmunity in response to osmotic stress were suppressed. These findings suggest that ACQOS translocates into the nucleus via the NPC, with NUP85, NUP96, and NUP107 playing an important role.

## Data availability statement

The datasets presented in this study can be found in online repositories. The names of the repository/repositories and accession number(s) can be found below: https://www.ddbj.nig.ac.jp/, DRA016084.

## Author contributions

KM: Conceptualization, Data curation, Formal analysis, Investigation, Writing – original draft. YM: Data curation, Formal analysis, Writing – review & editing. MT: Formal analysis, Writing – original draft. SK: Formal analysis, Writing – original draft. KN: Data curation, Formal analysis, Writing – review & editing. HA: Data curation, Writing – review & editing. KT: Data curation, Writing – review & editing. SI: Resources, Writing – review & editing. IY: Data curation, Writing – review & editing. YS: Data curation, Writing – review & editing. TT: Conceptualization, Funding acquisition, Supervision, Writing – original draft, Writing – review & editing.

## Supplementary material

Supplementary Figure 1 T-DNA insertion mutants of five *nup* mutants. **(A)** T-DNA insertion site of T-DNA insertion mutants. **(B)** Expression of *NUP85* in WT and *nup85* mutant by RT-PCR. The fragments were separated on an agarose gel and stained with ethidium bromide. Arabidopsis *ACTIN2* (*ACT2*) was used as the semiquantitative control.

Supplementary Figure 2 Expression levels of osmotic-, ABA- and immune-responsive genes in WT and *aot19* mutant under mild and severe osmotic stress. Expression of *RD29A*, *COR15A*, *COR47*, *ABI5*, *PR1*, *PR2* and *PR5* genes in WT and *aot19* plants under normal, mild (300 mM sorbitol for 5 h and 3 d) and severe (750 mM sorbitol for 5 h and 3 d) osmotic stress conditions. Expression levels were determined by qRT-PCR and were normalized to those of *Actin2*. Differences between WT and *aot19* plants were analyzed by Student’s *t*-test (mean ± SE, *n* = 3, **P* < 0.05).
