# Supplementary figures and images for: Mutations in nuclear pore complex promote osmotolerance in Arabidopsis by suppressing the nuclear translocation of ACQOS and its osmotically induced immunity
Source: Front Plant Sci. 2024 Jan 22;15:1304366. doi: 10.3389/fpls.2024.1304366 (PMC10839096; doi:10.3389/fpls.2024.1304366)

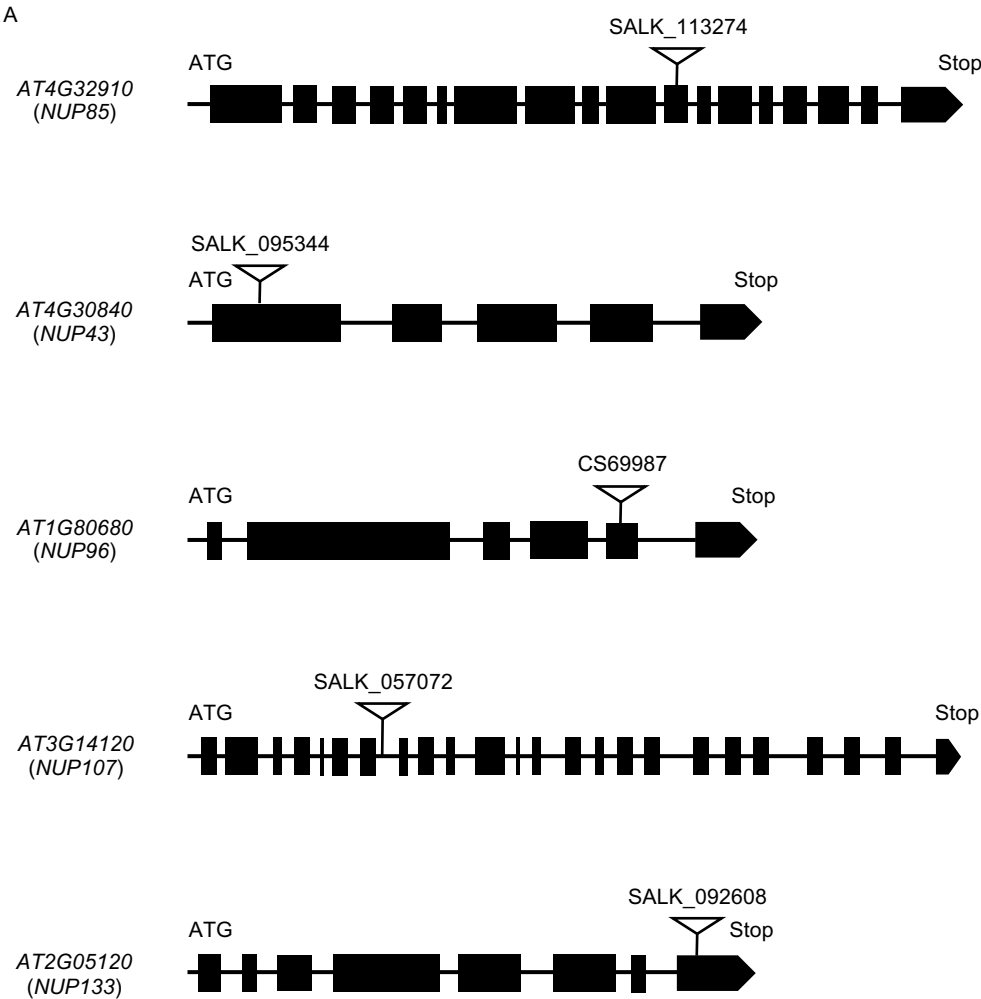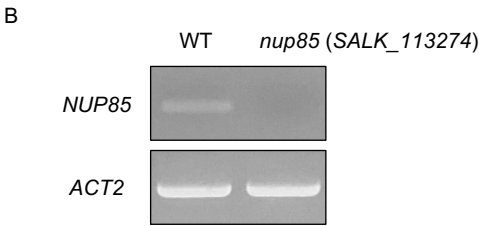

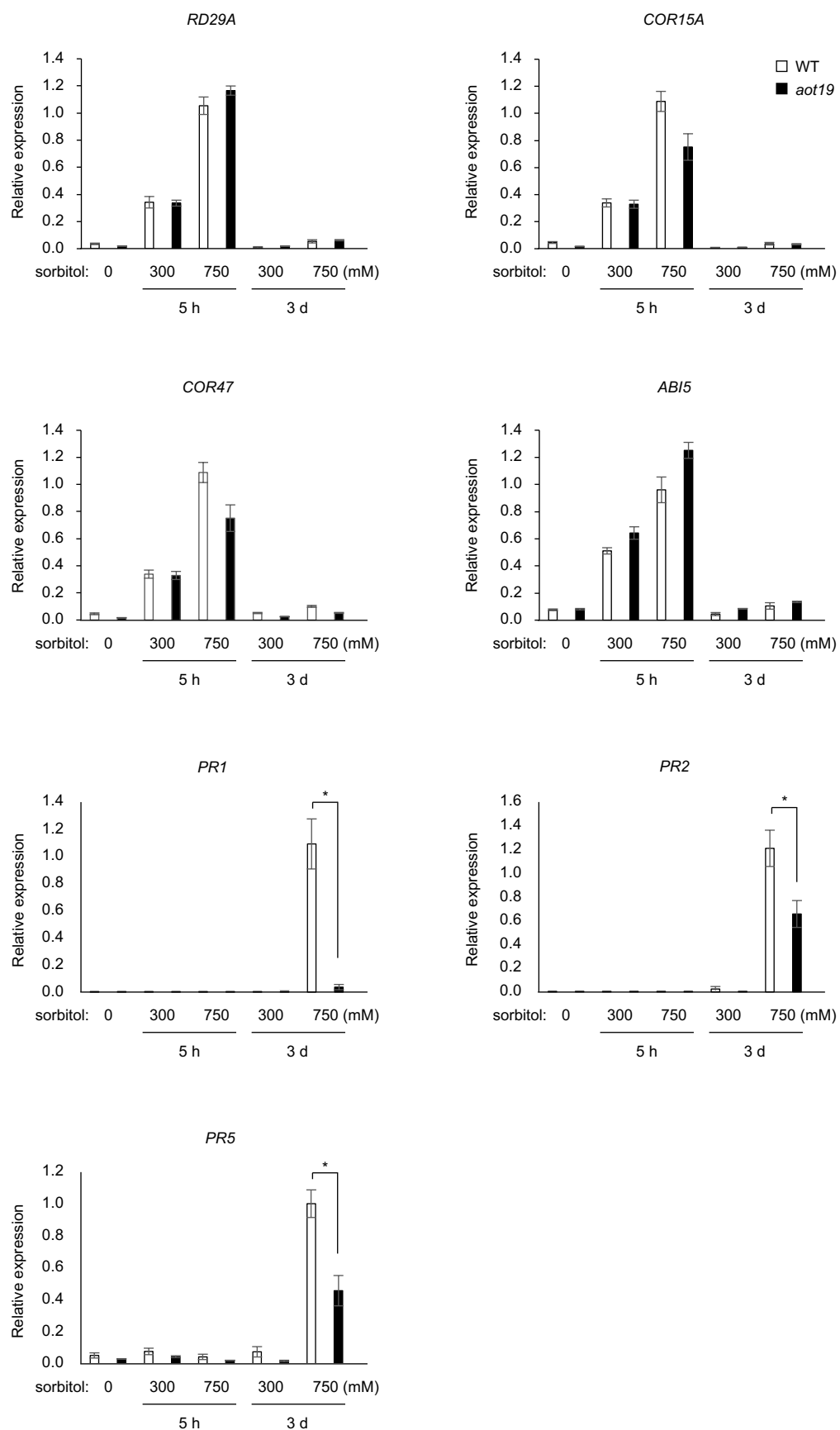

Supplement: Supplementary Figure 1 — T-DNA insertion mutants of five nup mutants. (A) T-DNA insertion site of T-DNA insertion mutants. (B) Expression of NUP85 in WT and nup85 mutant by RT-PCR. The fragments were separated on an agarose gel and stained with ethidium bromide. Arabidopsis ACTIN2 (ACT2) was used as the semiquantitative control. [file DataSheet_1.pdf]
