# Supplementary material for: Mutations in nuclear pore complex promote osmotolerance in Arabidopsis by suppressing the nuclear translocation of ACQOS and its osmotically induced immunity
Source: Front Plant Sci. 2024 Jan 22;15:1304366. doi: 10.3389/fpls.2024.1304366 (PMC10839096; doi:10.3389/fpls.2024.1304366)
Supplement: Supplementary file 2 [file DataSheet_2.pdf]

Table S1. Primers used for cloning, sequencing, genotyping, and qRT-PCR.

Name	Sequence	Purpose
salkLB	ATTTTGCCGATTTCGGAAC	T-DNA verification
nup43 (SALK_095344)F	ATCATTGGAAAATGAAAGGGC	T-DNA verification
nup43 (SALK_095344)R	AATCCATTCCCATCAAAGACC	T-DNA verification
nup85 (SALK_113274)F	ATGTTTCCAATGATGCACTCC	T-DNA verification
nup85 (SALK_113274)R	AATACCATTTTGCATTGCAGG	T-DNA verification
nup96 (CS69987)F	GATGCACGAAGCTCTGGTAAG	T-DNA verification
nup96 (CS69987)R	AACTTGGGCTGTGTTGTCATC	T-DNA verification
nup107 (SALK_057072)F	CCCATTGTCATTTTGGTATGC	T-DNA verification
nup107 (SALK_057072)R	GCAGAGATCACATGCCTCTTC	T-DNA verification
nup133 (SALK_092608)F	CATGCATTCCAATTTTATGCC	T-DNA verification
nup133 (SALK_092608)R	AATGGAGAAGCTAGAAACCGG	T-DNA verification
mos6 (SALK_119474)F	GCTTTCAATGCTGAGAAATGC	T-DNA verification
mos6 (SALK_119474)R	TCCAACCACGAGAATGTTCTC	T-DNA verification
229327 F	GTCCATAATTTTCATTGCTATTTC	Genetic mapping
229327 R	CAGGCATGGTTTTAAGGTAG	Genetic mapping
10049425 F	GAAGCAAGAGTTTTTTTTTTCTTC	Genetic mapping
1004925 R	CCAAAAGTTTTTGATCGG	Genetic mapping
13924584 F	GAAAGAGAGTTTACGGGGAAG	Genetic mapping
13924584 R	CTTTCACAACCAACAGTCTCG	Genetic mapping
14776616 F	GCTTGGATTTTTTAATTTATATTTGACTAAG	Genetic mapping
14776616 R	CTTTCTTTAAACAAAACCTAATAGGTGTC	Genetic mapping
15184838 F	CTCGGTACCAAACAAATCAG	Genetic mapping
15184838 R	CTGGTAATTTTTTATGCTCAAAGTTG	Genetic mapping
15489649 F	GCACAAAAAAATAACTTGCAC	Genetic mapping
15489649 R	CTGGACTAGAGATTATATCAATTTGTC	Genetic mapping
16022929 F	GAATGCATAAACCTTTTTTTAACTC	Genetic mapping
16022929 R	CAAATTTGGTTATTATCATCTTTTC	Genetic mapping
aot19 mutation (nup85) F	GCGAGTGAGGTATAGGTTTGA	Mutation detection
aot19 mutation (nup85) R	CTAACCTCACGATGTCCCAG	Mutation detection
At4g32910 infusion Fw	CGGTATCGATAAGCTAAGCTCTTGGTCCTTGCC	Vector construction
At4g32910 infusion Rv	ATTCGATATCAAGCTCTGCATTTCTCAGCAATCCAAC	Vector construction
AOT19 sequencing_1	AAGCTCTTGGTCCTTGCCAT	AOT19 sequencing
AOT19 sequencing_2	AAAAACATGATAAATTCTCG	AOT19 sequencing
AOT19 sequencing_3	TTGACAAAAAAGAAGCAACA	AOT19 sequencing
AOT19 sequencing_4	ATGCCGGGTATGTCTTCG	AOT19 sequencing
AOT19 sequencing_5	CAGGTGGGAGTACGTAATGG	AOT19 sequencing
AOT19 sequencing_6	TGTTGGCCTGCAGGTTTGCT	AOT19 sequencing
AOT19 sequencing_7	GCCACGAATTCTGCGGCCAC	AOT19 sequencing
AOT19 sequencing_8	CAAAGCCAGTTAACACTTCC	AOT19 sequencing
AOT19 sequencing_9	CACCTTTCTTATTGCGCTTC	AOT19 sequencing
AOT19 sequencing_10	GGGACGAAAATTGTCTCAGC	AOT19 sequencing
AOT19 sequencing_11	AATTACTGCAGCTAATGAAG	AOT19 sequencing
AOT19 sequencing_12	CAGAAAAGAGAGGTGTTCTG	AOT19 sequencing

AOT19 sequencing_13	TCTTTTTTAGATAGAGGAAT	AOT19 sequencing
ACQOS_exon1_F	CCAAAGTCTTCGTTTCTAGTT	35S:ACQOS-GFP construction
ACQOS_exon1_olPCR_R	TTCGTCATCCCATTTGGCAGAATCAAATCCA	35S:ACQOS-GFP construction
ACQOS_exon2_olPCR_F	CTGCCAAATGGGATGACGAAGCAAAAATGAT	35S:ACQOS-GFP construction
ACQOS_exon2_olPCR_R	TTCGAGTACCAATGCCTTCACTGAGTACAGT	35S:ACQOS-GFP construction
ACQOS_exon3_olPCR_F	TGAAGGCATTGGTACTCGAAAGGTGCTAGGA	35S:ACQOS-GFP construction
ACQOS_exon3_olPCR_R	ATGTAAATGACATAGCTCCTTCCCACAGCTT	35S:ACQOS-GFP construction
ACQOS_exon4_olPCR_F	AGGAGCTATGTCATTTACATGTCTAAAGGAA	35S:ACQOS-GFP construction
ACQOS_exon4_olPCR_R	GCATGAAGGGCTTCACTCCTTGGCATTTATT	35S:ACQOS-GFP construction
ACQOS_exon5_olPCR_F	AGGAGTGAAGCCCTTCATGCCGATGCTGTCT	35S:ACQOS-GFP construction
ACQOS_exon5_olPCR_R	AGTGGTGTAGCCGCATGCGCTTCTTGCTTCT	35S:ACQOS-GFP construction
ACQOS_exon6_olPCR_F	GCGCATGCGGCTACACCACTTCATATGAGTA	35S:ACQOS-GFP construction
ACQOS_exon6_R	CCTCGCTCTTCGCTCTGATCA	35S:ACQOS-GFP construction
ACQOS_CDS_F	ATGGCTTCTTCATCTTCTTCTCG	35S:ACQOS-GFP construction
ACQOS_CDS_R	TATGAAGTGGTGTAGCCGCATGC	35S:ACQOS-GFP construction
aot19 intron junction F	CGTGGGAGTTGATGGAATTA	RT-PCR
aot19 intron junction R	GGATGTGAGCTAGAAAGGAG	RT-PCR
NUP85 F	CTAGAGATTTGCCGTCTCTATGAAC	RT-PCR
NUP85 R	CTGTTGAGCAATCACGCTG	RT-PCR
ACT2 RT F	ATCTTCTTCCGCTCTTTCTTTCCA	RT-PCR
ACT2 RT R	CATAAAACCCCAGCTTTTTAAGCC	RT-PCR
ACT2 qRT F	ACCTTGCTGGACGTGACCTTACTGAT	qRT-PCR
ACT2 qRT R	GTTGTCTCGTGGATTCCAGCAGCTT	qRT-PCR
PR1 qRT F	GTGGGACGAGAGGGTTGCAGCCTAT	qRT-PCR
PR1 qRT R	GCACGTGTTCGCAGCGTAGTTGT	qRT-PCR
PR2 qRT F	CAGATTCCGGTACATCAACGTT	qRT-PCR
PR2 qRT R	AGTGGTGGTGTCAGTGGCTA	qRT-PCR
ACQOS qRT F	GAAGCGCATGCGGCTACACCACTT	qRT-PCR
ACQOS qRT R	GTTCAGTCACATTGCCTATACCAT	qRT-PCR
RAB18 qRT F	CCGTTAAGCTTCGAACAATCGTGT	qRT-PCR
RAB18 qRT R	CAACACACATCGCAGGACGTACA	qRT-PCR
COR15A qRT F	GAGGCATTAGCAGATGGTGAGA	qRT-PCR
COR15A qRT R	CCACATACGCCGCAGCTTTCT	qRT-PCR
RD29A qRT F	GGCGTAACAGGTAAACCTAGAG	qRT-PCR
RD29A qRT R	TCCGATGTAAACGTCGTCC	qRT-PCR
KIN1 qRT F	GGAAGGCATTCTTGTTGGTCTCTG	qRT-PCR
KIN1 qRT R	GCCCACATCTCTTCTCATCATCAC	qRT-PCR
COR47 qRT F	ACAAGCCTAGTGTCATCGAAAAGC	qRT-PCR
COR47 qRT R	TCTTCATCGCTCGAAGAGGAAG	qRT-PCR
ABI5 qRT F	CGGGTTTGGATTAGGTTTAG	qRT-PCR
ABI5 qRT R	GTAGTAGTAGTAGTAATGGACAGA	qRT-PCR
PR5 qRT F	TAGCTACGCTTATGACGACGAA	qRT-PCR
PR5 qRT R	ACCCGACTGTATCTAACTCGAAGC	qRT-PCR
